# Clinical symptoms and sonographic follow-up after surgical treatment of nonparasitic liver cysts

**DOI:** 10.1186/1471-2482-13-42

**Published:** 2013-09-30

**Authors:** Hubert Scheuerlein, Falk Rauchfuss, Julia Franke, Karin Jandt, Yves Dittmar, Gudrun Trebing, Utz Settmacher

**Affiliations:** 1 Department of General, Visceral and Vascular Surgery, University Hospital Jena, Erlanger Allee 101, 07747 Jena, Germany

## Abstract

**Background:**

The optimal treatment of nonparasitic liver cysts is still a topic of debate. Only symptomatic cysts are being considered as requiring treatment. Aim of this study is to evaluate our experience with this disease over the past ten years with a structured follow-up program.

**Methods:**

From January 2000 to August 2010, 56 consecutive patients with nonparasitic liver cysts were treated at our institution. We assessed morbidity, recurrence and complication rates, quality of life as well as pre- and post-operative sonographic status of the cysts and course of clinical symptoms.

**Results:**

In 84% of the patients surgery was started as a laparoscopic procedure. Conversion rate was 6.4%. Average diameter of deroofed cysts was 12 cm. Overall complication rate was 16% and overall recurrence rate 28.3% (8.7% recurrences at the surgical site, 19.6% new or enlarged cysts). One half of the patients were symptom-free after surgery and the other half had at least one persisting symptom post-operatively. In one half of these patients with persisting symptoms, symptoms were ameliorated by surgery. In the other half of patients the number of symptoms increased after surgery. Two thirds of the overall patients reported their post-operative health as being good or very good.

**Conclusions:**

Surgical deroofing is the most effective treatment option for symptomatic liver cysts. Half of our patient population retained at least one symptom from a group of more than ten abdominal symptoms.

Only the minority of these cases may be attributed to true recurrence, de-novo cysts or growing pre-existing cysts. The analysis of our cases suggests that the persistent symptoms in our patients may in part be due to the fact that the association between clinical complaints and the liver cysts was not sufficiently established. A more rigid patient selection should be implemented in order to achieve better results from the treatment of cysts. Because even large cysts are frequently asymptomatic, patient selection should not primarily be based on the cyst size only. The decision should be based strictly on the correlation between cyst / cyst location and symptoms / clinical complaints. In our opinion, further diagnostic procedures may be necessary in individual cases to clarify such a correlation.

## Background

Nonparasitic liver cysts occur in up to 5% of the population [1,2]. There are simple (single or multiple) cysts (simple liver cyst, SLC) as opposed to polycystic liver disease (PCLD). There is currently a general agreement that only symptomatic cysts should be treated. 10%-16% of patients with liver cysts develop symptoms at some stage in life [3]. Symptoms develop at an advanced age and are non-specific. Women are more frequently affected [4,5]. As the recurrence rate of percutaneous drainage alone is up to 100%, this procedure cannot be accepted as the only treatment [6]. For sclerotherapy very heterogeneous success rates between 0% and 100% are being reported with a low complication rate and short hospital stay [7]. In a recent prospective sclerotherapy study in 20 patients with 20% saline the cysts had completely disappeared after 2 years follow-up in 40% of the patients [8]. There are no larger or randomized comparative studies. Thus, sclerotherapy cannot be evaluated sufficiently with respect to its definitive value as yet. Laparoscopic cyst deroofing is the gold standard for treatment if this is feasible with respect to the location and configuration of the cysts [9,10]. Its value, too, has not been definitively assessed, but there are data that the short-term outcomes of laparoscopic fenestration were superior to the open approach [11]. Very heterogeneous success rates between 43% and 96% are being reported [2]. Irrespective of the surgical methods, true recurrence and growing remnant cysts are frequently reported in the literature. There is no definitive correlation with clinical symptoms. Studies with a larger sample size (> 50) and/or specific pre- and post-operative comparison of symptoms are scarce [2,11]. The value of this present study lies in its systematic sonografic and clinical follow-up after surgical cyst deroofing with a large patient population and long follow-up period.

## Methods

Between January 2000 and August 2008, 56 consecutive patients with symptomatic nonparasitic hepatic cysts were evaluated and treated at our institution.

The decision for surgery was generally based on a minimum cyst size of 5 cm and abdominal pain, dyspnea and/or vegetative symptoms caused by adjacent organ compression or increased abdominal pressure and, therefore, causing gastrointestinal transport impairments, resulting in nausea, vomiting, oesophageal reflux, loss of appetite and/or early satiety. Mainly cysts located on the liver margin or underneath the surface within the parenchyma of the segments II, III, IVb, V and VI were selected for laparoscopic surgery.

There were 49 women and 7 men, with a mean age of 59 years (range 39 to 81 years). 4 patients had PCLD. All patients underwent pre-operative sonography. The echinococcus serology was negative in all cases.

These were the criteria for sonographic evaluation as benign / non-parasitic liver cyst: smooth margins, no septum, watery / clear cyst fluid, no contrast enhancement and calcifications. Further diagnostic procedures were initiated in cases of even the slightest suspicion of parasitic or neoplastic cyst.

All operations were performed as deroofing. The decision for open or laparoscopic surgery was made by the respective surgeon depending on his/her experience, cyst location and relevant co-morbidities (e.g. vena cava thrombosis being a contra-indication against the laparoscopic technique). The decision whether to perform an omentoplasty was also made exclusively by the operating surgeon. For open surgery, access was gained via a J incision. Laparoscopic surgery was performed via a 3-trocar-technique in the region of the right hypochondrium (in individual cases, another trocar was used as auxiliary trocar). For deroofing, we attempted to remove a maximum of the cyst roof ("wide deroofing"). The operating surgeon decided on the dissection method and in most cases, ultrasonic dissection was performed (Ultracision harmonic scalpel, Ethicon Endo Surgery, Norderstedt, Germany).

A specific questionnaire with regard to medical history (including a detailed history of the medication, paying special attention to female sexual hormones), symptoms and quality of life was developed. The patients were interviewed post-operatively and were asked to compare their current clinical complaints with the pre-operative complaints. All patients had additionally been invited for a sonographic and clinical follow-up examination. The pre- and post-operative sonographic examination was standardized on the basis of the Couinaud segment classification. Statistics were performed with SPSS 18 (chi-square test, Kruskal-Wallis test, paired t-test, Wilcoxon test). Patients were analysed regarding demographics, cyst morphology, symptoms and complaints, therapeutic procedures, postoperative complications according to Clavien-Dindo classification and longterm results (synopsis of relevant demographic and clinical characteristics in Table 1).

**Table 1:** Demographic and clinical characteristics

	
Male : Female	7:49
Simple liver cysts : Polycystic liver disease	52:4
Age, years (range)	59 (39–81)
BMI, kg/m^2^ (range)	27 (19–41)
ASA I, n (%)	9 (16)
ASA II, n (%)	42 (75)
ASA III, n (%)	5 (9)
Diameter of dominant cyst, cm (range)	12 (6–20)
Laparoscopic/open deroofing, n (%)	47 (84)/9 (16)
Conversion rate (%)	6.4
Simultaneous cholecystectomy, n (%)	16 (29)*
Postoperative complication rate (%)	16**
Recurrence rate (%)	8.7

*8/47 (17%) laparoscopic, 8/9 (89%) open; **13% laparoscopic, 33% open, not significant.

*Abbreviations*: *BMI* Body Mass Index, *ASA* American Society of Anesthesiologists.

An approval of the local ethic’s committee was not needed in this design, since all patients give their consent to follow-up investigations and further use of their data when entering our department. Furthermore, the study design was non-invasive.

## Results

### General aspects

For pre-operative work-up, 77% of patients had computed tomography in addition to sonography and 5% had an MRI.

Complete follow-up (sonography, clinical examination and complete questionnaire) was achieved in 46/51 patients (90%). The average follow-up period was 43 months (3–119 months). Four patients had died during follow-up (the cause of death was not related to the liver cyst or surgery for liver cysts) and one patient with PCLD had a liver transplantation.

The cysts had been known for 40 months on average (0–360 months). The average BMI was 27 kg/m^2^ (19–41 kg/m^2^). 46/49 women (94%) had given birth. Use of female sexual hormones postmenopausal or for contraception was recorded in 22% and 67% of patients, there was no significant association with symptoms or cyst size. All surgical samples were examined by the pathologist and there was no malignancy. 41/56 (73%) did have prior abdominal surgery and 24/49 (49%) female patients did have prior gynaecological surgery. 18/56 (32%) patients did have other concomitant diseases of the gastrointestinal tract (Table 2). As for non-abdominal co-morbidities, these were mainly cardiac/circulatory (43%) and metabolic (32%). 16/56 (29%) patients had a simultaneous cholecystectomy (6× gall stones, 9× close proximity to the cyst, 1 both factors). 7/56 (13%) patients underwent previous treatment for their cysts elsewhere (5 percutaneous drainages, 1 laparoscopic and 1 open cyst fenestration). 9/56 (16%) patients were ASA class I, 42/56 (75%) were ASA class II and 5/56 (9%) were ASA class III. There was no surgical mortality.

**Table 2:** Concurrent diseases of the gastrointestinal tract (multiple answers permitted)

**Disease**	**n**
Gallstones	8
Diverticulosis of the sigmoid colon	4
Steatosis hepatis	3
GERD	3
Gastritis	3
Peptic ulcer	1
Exocrine pancreatic insufficiency	1

GERD – Gastroesophageal reflux disease.

### Pre-operative symptoms

The distribution of pre-operative symptoms is shown in Table 3 (multiple answers possible, minimum number of symptoms 1, maximum number of symptoms 7).

**Table 3:** Pre-operative symptoms (multiple answers permitted)

**Symptom**	**n**	**%**
Upper abdominal pain	33	58.9
Feeling of pressure	32	57,1
Fullness	8	14,3
Nausea	8	14,3
Weight loss	3	5,4
Vomiting	3	5,4
Dyspnoea	3	5,4
Early satiety	2	3,6
Loss of appetite	2	3,6

### Cyst status

17/52 (33%) patients with SLC had single and 35/52 (67%) had multiple cysts. Of the 4 patients with PCLD 2 had Gigot stage I and 2 had Gigot stage III. Only the left or the right liver lobe was affected in 7 (13%) and 11 (20%) cases, respectively. In the remaining 38 patients, both lobes were affected. There was a cyst related hepatomegaly in 41% of the patients. The average diameter of the deroofed cysts was 12 cm (6–20 cm). In one third of the patients, the segments IVa, VII and VIII were affected (these are considered as being difficult to access via the laparoscopic approach).

### Laparoscopic surgery

In 47/56 (84%) patients surgery was started as a laparoscopic procedure. In 3 patients, there was a conversion to open surgery (conversion rate 6.4%). The reasons for conversion were hemorrhage (2 cases) and an incidental finding of a peritoneal metastasis from breast cancer (1 case). The average surgical duration was 80 min (16–230 min). In cases of simultaneous cholecystectomy (8 cases) the surgical duration was significantly prolonged, on average by 20 min (p = 0.014). A mean number of 2 cysts (1–9 cysts) were deroofed. In 5 patients, omentoplasty was performed. The average post-operative hospital stay was 6 days (4–13 days) following deroofing and cholecystectomy and 5.2 days (2–19 days) after deroofing only, respectively. The difference was not significant (p = 0.291).

### Laparotomy

9/56 patients (including 2/4 patients with PCLD, both stage Gigot I) have had a primary laparotomy. The reasons for primary laparotomy included: cyst-related inferior vena cava thrombosis (2 cases), massive cystic liver (1 case), recurrence (1 case), previous Billroth-II resection (1 case), abscess (1 case) and position or morphology of the cysts deemed unfavourable (3 cases). The mean surgical duration was 106 min (44–173 min). An average of 2 cysts (1–3 cysts) have been fenestrated. Omentoplasty was performed in 3 cases. A simultaneous cholecystectomy was performed in 8 patients. When compared to the laparoscopic procedure, the post-operative hospital stay was significantly longer (mean 8.3 days, range 6–21 days; p = 0.004).

### Complications

#### ***Intraoperative complications***

There were 8 intra-operative complications in the laparoscopic group. These were injuries to the liver parenchyma (4 cases), one injury to the left hepatic vein, one hemorrhage during omentoplasty, one bile leak and one injury to the adrenal gland. The injury to the hepatic vein and one parenchymal hemorrhage led to conversion, all others were managed laparoscopically. There were no complications in the laparotomy group. Thus, the total intra-operative complication rate was 14% (8/56; laparoscopy group 8/47 (17%), laparotomy group 0/9 (0%)). The intra-operative complication rate was not significantly different (p = 0.361).

#### ***Postoperative complications***

The total post-operative complication rate was 16% (9/56; laparoscopy group 6/47 (13%), laparotomy group 3/9 (33%)). There was a total of 14 complications, including 10 complications according to the Clavien-Dindo classification I (5 pleural effusions; ascites, impaired wound healing, partial respiratory insufficiency, dyspnoea, fever, one case each), one Clavien-Dindo classification II (urinary tract infection) and 3 cases of Clavien-Dindo classification IIIa (bilioma / bile leak). There was a significant difference between the two surgical techniques (p = 0.031).

### Follow-up

#### ***Immediate post-operative sonography***

In 50/56 (89%) patients, a sonographic examination was performed immediately after surgery. In 29/50 (58%) patients, there were no more cysts, in 21/50 (42%) patients, remaining cysts have been observed. The total cysts volume had been reduced on average by 47% (6%-85%) through surgery in patients with remnant cysts.

#### ***Sonographic follow-up examination***

The mean follow-up period was 43 months (3–119 months). 46/56 (82%) patients had a sonographic follow-up examination. Only 2/46 (4%) patients had no sonographic evidence of cysts. 11/46 (24%) patients had single and 33/46 (72%) patients had multiple cysts (including 3 patients with PCLD). There was cyst-related hepatomegaly in 15/46 (33%) patients. Only 3 patients with remaining cysts did not have any clinical complaints. In 8 patients, the size of the cysts had increased as compared to the immediate post-operative examination.

#### ***Symptoms and recurrence rate during follow-up***

23/46 (50%) patients were completely symptom-free and 23/46 (50%) patients had persisting symptoms. 3/23 (13%) patients with persisting symptoms had no symptom change in the pre-/post-operative comparison, whereas 12/23 (52%) patients had increasing and 8/23 (35%) patients decreasing symptoms (Table 4). The post-operative symptoms included feeling of pressure (33%), upper abdominal pain (24%), fullness (15%), dyspnoea (15%), nausea (13%), early satiety (11%) and weight loss (2%; multiple answers possible). In 3/8 (38%) of the patients with decreased symptoms there was probably an association with a new cyst (5 cm, 7 cm and 11 cm, respectively). In 7/12 (58%) of the patients with increased symptoms relevant cysts have been observed (5–20 cm, average cyst size 9 cm). In one of these patients the increasing clinical complaints were most likely caused by a symptomatic incisional hernia. In the 3 patients with identical symptoms pre- and post-operatively, there might have been an association with a new cyst of 5 cm in size and in one case this was probable for a cyst of 15.5 cm. Therefore in altogether 12/23 (52%) patients with persisting or increasing symptoms this may generally be explained by a new cyst (8 of these 12 patients with a new cyst had a cyst size of at least 6 cm). Recurrences developed in 13/46 (28.3%) patients. 4 of those (4/46, 8.7%) have been "true surgical" recurrences at the former surgical site. All recurrences occurred after laparoscopic fenestration. There was, however, no significant association between recurrence and surgical technique (p = 0.562). 3 patients did have further surgery for liver cysts during the follow-up period. There was one open surgical revision (adhesions/stenosis of the gastro-duodenal junction) and 2 laparoscopic re-operations. The Kruskal-Wallis test did not show any difference between the surgical techniques with respect to the number of reported clinical complaints. The decrease of the number of symptoms post-operatively as compared to pre-operatively was significant after laparoscopic surgery (p = 0.004) but did not improve after open or conversion surgery (p = 0.667 and p = 1.000, respectively).

**Table 4:** Symptom change in patients with persisting symptoms

**Symptom change**	**Number of symptoms**	**Δ (Difference)**
	**Pre-operative** → **Post-operative**	
No change (n = 3)	2 → 2	0
	1 → 1	0
	1 → 1	0
Symptom increase (n = 12)	2 → 6	4
	4 → 7	3
	1 → 4	3
	1 → 3	2
	1 → 3	2
	1 → 3	2
	1 → 3	2
	2 → 3	1
	2 → 3	1
	1 → 2	1
	1 → 2	1
	1 → 2	1
Symptom decrease (n = 8)	5 → 1	4
	4 → 2	2
	6 → 5	1
	3 → 2	1
	2 → 1	1
	2 → 1	1
	2 → 1	1
	2 → 1	1

#### ***Symptoms and description of the quality of life***

7/46 (15%) patients reported their current health condition as being very good while 24/46 (52%) patients reported it as being good. 15/46 (33%) reported their current health condition as constricted, 13 of these 15 patients felt impaired in their daily lives by the reported symptoms. On a scale from 0–10, 33/46 (72%) patients did not report any impairment (0). 6/46 (13%) patients reported minor impairment (grade 1–3) and 7/46 (15%) patients reported moderate to major impairment (grade 4–8). 13/33 (39%) patients with an impairment degree of 0 in their daily lives did report at least 1 symptom in their history of clinical complaints.

## Discussion

For SLCs cyst deroofing (laparoscopic wherever possible) is considered as being the therapeutic gold standard. The symptoms in patients with liver cysts are non-specific. Die Zystengröße allein scheint eine untergeordnete Rolle zu spielen, da selbst bei großen kongenitalen Leberzysten asymtpmatische Verläufe keine Seltenheit sind [3]. It is certain that other factors play a role, in particular the location of the cysts. So far, in the literature, there are hardly any considerations of psychosomatic factors that may play a role in a certain patient proportion which may be difficult to identify; these factors are complex and difficult to measure.

For reasons of clarity of the terminology and because of the fact that too small a fenestration may favour recurrence, the term complete cyst *deroofing* should be used in order to emphasize this aspect [3,12].

The value of the current study is in its rather concise follow-up (clinical examination, sonography, questionnaire) in a sufficiently large patient population and long follow-up period. Our patient population is comparable to those of other groups on the topic with respect to age, gender distribution, cyst size, symptoms and complication rate [2,10,13]. Studies with more than 50 patients and a specific follow-up are scarce [2,10,11,13,14]. A sufficiently long follow-up period is of critical importance in patients with liver cysts because there is evidence that a relatively large patient proportion (5-50% according to the literature) develops recurrent clinical symptoms from cysts within 3–5 years [2]. The mean follow-up period in this current study is 43 months. This duration was reached or surpassed in only half of the studies published so far [2]. There are only scarce exact comparisons of pre- and post-operative symptoms in the literature [2,11].

Our altogether recurrence rate is as high as 28.3%. For principal reasons in our opinion the type of recurrence (i.e. a "true surgical recurrence" at the former surgical site vs. a "recurrence of the disease" in terms of completely new or enlarged pre-existing cysts) should be considered as different entities in an analysis of surgical outcome. This discrimination can only be made by an accurate imaging during the follow-up and will not be done in the daily routine. Split like this our surgical recurrence rate was 8.7% and recurrent disease occurred in 19.6%, both rates being independent of the surgical approach.

Our study includes 4 patients with PCLD. Cyst deroofing (possibly laparoscopically) in Gigot stage I may provide a long lasting improvement. The recurrence rate in PCLD is generally higher, and more frequently liver resection is the treatment of choice [2]. Thus, there is a fundamental difference between SLC/PCLD stage I and PCLD stage II/III. In cases of diffuse disease, reduced nutritional status, extreme hepatomegaly and ascites, liver transplantation may be considered (in cases of GFR < 40 ml/min with simultaneous kidney transplantation) [15].

As in selected patients with PCLD the decision for resection or deroofing may be made, these cases were primarily included in the analysis. However, numerous reports in the literature suggest that patients with PCLD should be analyzed separately. Even though our 4 patients with PCLD should not have falsified our results significantly, we would strictly separate these two entities in future studies.

In all studies, females predominate (1:3 – 1:9) [2,10,12,13]. The age peak is around 60 years, as it is in our patient population. Between the age of 30 and 60 years, there is a progressive growth of cysts for reasons that are not known so far [16]. Several authors have linked this to the influence of estrogens [17]. In our patient population, we did not observe a significant association between female sexual hormones and symptoms or cyst size.

16/56 (29%) of our patients underwent simultaneous cholecystectomy for concomitant cholecystolithiasis or immediate proximity of the gallbladder to the cyst (8 open, 8 laparoscopically; 6 concomitant cholecystolithiasis, 9 immediate proximity, 1 concomitant cholecystolithiasis and immediate proximity).

The decision for surgery was based on the liver cyst even though it is not possible to clarify definitively if in case of concomitant gall stones the reported symptoms may be attributed to these (none of these patients did show typical signs of gall stone related disease, such as relevant elevated laboratory parameters, sonographic criteria or typical symptoms in the medical history). In case of gall stones, we generally perform a cholecystectomy simultaneously at our institution, except in cases where the patients decides otherwise.

In 84% of the patients, the cyst deroofing was started as a laparoscopic procedure, the conversion rate was 6.4%. There was previous abdominal or gynaecological surgery in 73% and 49%, respectively, while only in one case the previous abdominal surgery (Billroth-II resection) was the reason for a primary open procedure. In a total of 9 cases, the surgical procedure was primarily open (3 unfavorable cyst locations, 2 vena cava thromboses, 1 PCLD, 1 recurrence, 1 abscess, 1 Billroth-II resection). As reported in the literature, this approach was reserved for particular situations. In both cases of a concomitant vena cava thrombosis, successful thrombectomy was performed.

Three fourths of the patients were ASA stage II and almost one tenth were stage III. This explains the comparatively long hospital stay of 6 and 8 days, respectively (laparoscopic and open group, respectively).

In the literature, there are occasional reports about predominant affection of the right or left lobe. We could not confirm that observation in our patient population. In one third of our patients with laparoscopic surgery, segments IVa, VII and VIII were affected; access to these is considered to be difficult. This is as such not a contraindication against the laparoscopic approach, neither did it affect the conversion rate in a negative way. We believe that this confirms the experience of other authors, that this problem is met by an on-going learning curve on one hand and by specific patient positioning (modified left sided position) as required on the other hand.

In the laparoscopy group, the intra-operative complication rate was 17% (possibly related to the learning curve) while it was 0% in the laparotomy group (difference not significant). Those were mainly bleeding complications which led to conversion in 2 cases. The post-operative complication rate of 16% was within the data reported in the literature. 3 cases (Clavien-Dindo grade IIIa) required intervention for bile leakage (sonographically guided drainage and/or ERC). The difference in the post-operative complication rate (laparoscopy vs. laparotomy 13% vs. 33%) was significant but does reflect in general the selection of "more difficult" patients for open surgery.

The use of omentoplasty is being discussed controversially in the literature. We have employed it in 14% of our patient population (8/56; 5 laparoscopically, 3 open). None of our patients with omentoplasty developed a recurrence. However, in one case, there was intra-operative bleeding from the omentum majus during laparoscopic omentoplasty; this did not necessitate conversion. Because of the few cases of omentoplasty in our patient population, we cannot make a definitive statement. There are reports about recurrence in the literature despite omentoplasty [10,18]. Furthermore, there is evidence that omentoplasty may cause additional complications or favour recurrence [10,19]. The data in the literature are controversial and there as many proponents as opponents of omentoplasty [2,9,10,18-21] while in more recent publications the value of omentoplasty is being viewed critically [10,18,19,21]. Currently, a falciform ligament pedicle graft is being proposed as an alternative prophylaxis against recurrence, but there are no long-term data available so far [22].

Half of our patient population (23/46, 50%) were completely symptom-free after cyst deroofing. The other half reported persisting symptoms. Only in some of these patients a new cyst is the explanation for the reported complaints.

This high rate may be explained by the design of the interview. 11 symptoms, possibly related to liver cysts, have been included in the interview. As soon as even only one of the 11 possible symptoms was reported in the post-operative interview, this was declared as persistent clinical complaints. In retrospect, this interview design is un-specific. Finally, the liberal decision for surgery may be an explanation as symptoms which may have had other causes may have not been decisively clarified and may have been falsely attributed to liver cysts. It may be beneficial to assess the psychosomatic aspects separately in order to relate them to the respective somatic complaints.

Our work also suggests that un-specific symptoms may have been judged too un-critically as being the cause of clinical complaints and, thus, have been judged to be relevant for the decision making (Table 3). The association between cyst location and the specific complaints should possibly have been established more precisely and only a very obvious association should lead to surgery (e.g. a relatively large cyst exerts pressure on the gastro-duodenal junction). We will, thus, perform more intensive diagnostics (in particular endoscopically) of the vicinity in the future in order to rule out concomitant disease. Furthermore, we consider the introduction of the cyst aspiration test in the future for better patient selection.

Irrespective of the accuracy of the follow-up, a recurrence rate of approximately 5% and an associated reoperation rate of less than 10% may be assumed [2,23,24]. As opposed to earlier reports in the literature [3,25], our data and numerous other studies show, that the laparoscopic procedure as such is no longer a risk factor for a higher probability of recurrence [2,11,13]. Carrying a low rate of complications and conversions, it is, thus, to be considered the treatment of choice for the vast majority of cases. In our study, we have mainly used the ultrasound dissector in the laparoscopically treated patients. Several authors see a potential association of the recurrence rate with the dissection technique. However, the development of recurrence is supposedly more affected by the extent of the fenestration/deroofing (and, thus, only indirectly by the dissection technique) [10,26].

Several groups report the avoidance of cyst recurrence after an ablation of the cyst lining in the residual fenestrated cystic cavity with argon beam coagulation [25,27]. We have used this technique so far only for liver resections. The results with argon beam coagulation to the cyst lining in the literature appear to be promising so that we will use it in the future in order to avoid cyst recurrence.

## Conclusions

In conclusion, our results, in context with the data from the literature may be summed up as follows: only symptomatic cysts should be operated. True/symptomatic recurrence develops in 5% to maximally 10% of patients. Recurrences will become most probably symptomatic again. In approximately one fifth of patients new cysts develop or existing cysts grow. It is currently not clear under which circumstances such conditions become symptomatic. In our study in one half of the patients the symptoms continued at varying degrees. In order to address this point appropriately, a more extensive diagnostic work-up and a meticulous patient selection are necessary in our hands. With respect to QoL, our results provide only very general information. Two thirds of the overall patients reported their post-operative health as being good or very good.

So far, the literature does not distinguish strictly between true recurrence (i.e. a new cyst exactly at the location where surgery was performed) and remaining cysts that grow in due course or de-novo symptomatic cysts. For the sake of better comparability of study results, a standardized follow-up (including sonography on a regular basis) as well as a sufficiently long follow-up period of at least 3 years are required. In adequately selected patients, laparoscopic (or open) deroofing is nevertheless the most effective treatment option for symptomatic patients. Our data may be limited by the retrospective character of our analysis. The above mentioned questions and particularities require further scientific evaluation.

## Competing interests

The authors declare that they have no competing interests.

## Authors’ contributions

HS collected the data, analysed the data and wrote the manuscript; FR analysed the data and wrote the manuscript; JF collected the data, analysed the data and wrote the manuscript; KJ analysed the data and wrote the manuscript; YD analysed the data and wrote the manuscript; GT performed the ultrasound investigations, collected the data and wrote the manuscript; US analysed the data and wrote and revised the manuscript. All authors read and approved the final manuscript.

## Pre-publication history

The pre-publication history for this paper can be accessed here:

http://www.biomedcentral.com/1471-2482/13/42/prepub
